# Neurological manifestations in Wiskott–Aldrich syndrome: a systematic review

**DOI:** 10.3389/fimmu.2026.1829058

**Published:** 2026-05-07

**Authors:** Nicholas Giulio Raccagni, Viktor Franco Milanesi, Giovanna Lucchini, Adriana Balduzzi, Giuseppe Occhino, Pietro Invernizzi, Serena Gasperini

**Affiliations:** 1 Department of Medicine and Surgery, Università degli Studi Milano-Bicocca, Monza, Italy; 2 Paediatric Clinics, Fondazione IRCCS San Gerardo dei Tintori, Monza, Italy; 3 Biostatistics and Clinical Epidemiology, Fondazione IRCCS San Gerardo dei Tintori, Monza, Italy; 4 Division of Gastroenterology, Center for Autoimmune Liver Diseases, European Reference Network on Hepatological Diseases (ERN RARE-LIVER), Fondazione IRCCS San Gerardo dei Tintori, Monza, Italy

**Keywords:** autoimmunity, immunodeficiency, neurological manifestations, rare disease, systematic review, thrombocytopenia, WAS gene, Wiskott–Aldrich syndrome

## Abstract

**Introduction:**

Neurological involvement in Wiskott–Aldrich syndrome (WAS) – an inborn error of immunity caused by mutations in the *WAS* gene – is primarily documented through isolated case reports, limiting systematic synthesis and clear characterization of clinical patterns. We conducted a systematic review to characterize central and peripheral nervous system involvement in patients with confirmed WAS.

**Methods:**

A systematic search was conducted in PubMed, Embase, Scopus, and Web of Science following the PRISMA 2020 guidelines. Studies reporting neurological manifestations in WAS were eligible. Extracted variables included neurological diagnosis, age at WAS diagnosis, age at neurological onset, hematopoietic stem cell transplantation (HSCT) status, viral associations, and outcomes. Methodological quality was assessed using the Newcastle–Ottawa Scale and Joanna Briggs Institute tools. Analyses were descriptive at the patient level.

**Results:**

Twenty-six studies describing 32 patients were included. Most patients were pediatric (78.1%), with a median age at WAS diagnosis of 0.4 years; neurological manifestations occurred a median of 3.0 years later. Manifestations were classified as brain hemorrhagic (8/32), immune-mediated (6/32), infectious (6/32), or neoplastic (12/32). Median age at neurological onset differed across categories (p = 0.018): brain hemorrhagic events occurred earliest (1.2 years), immune-mediated events in childhood (3.8 years), infectious events later (14.5 years), and neoplastic events across a broad age range (5.0 years). Infectious cases were predominantly John Cunningham virus–positive progressive multifocal leukoencephalopathy; neoplastic cases involved central nervous system lymphoma or post-transplant lymphoproliferative disorder. Case-fatality varied by phenotype (p = 0.002), reaching 100% in infectious, 75% in neoplastic, 62.5% in hemorrhagic, and 0% in immune-mediated cases. Overall, neurological event–attributed case-fatality was 59.4%. Events occurred both before and after HSCT, with numerically higher mortality among non-transplant patients (63.6% vs 50.0%).

**Discussion:**

Neurological involvement in WAS exhibits age-dependent phenotypic patterns, with substantial case-fatality particularly in infectious and neoplastic presentations. Although derived from case-based evidence, these findings support heightened neurological vigilance across the disease course and the need for systematic neurological reporting in future registries.

**Systematic review registration:**

https://www.crd.york.ac.uk/prospero/display_record.php?RecordID=1141002, identifier CRD420251141002.

## 1 Introduction

Wiskott–Aldrich syndrome (WAS) is a rare X-linked primary immunodeficiency caused by pathogenic variants in the *WAS gene* (1). In the 2024 International Union of Immunological Societies classification, *WAS* is included among the 508 genes known to underlie inborn errors of immunity (IEI), with loss-of-function and gain-of-function variants associated with distinct clinical phenotypes (2, 3). Accordingly, genetics-focused studies have linked variant class, mutation distribution, and genotype to disease expression, severity, and survival (4).

WAS is classically characterized by microthrombocytopenia, eczema, and combined immunodeficiency (5). Its clinical spectrum, however, extends beyond this triad to include hyperinflammatory complications such as hemophagocytic lymphohistiocytosis (HLH), often associated with Epstein–Barr virus (EBV) and frequently accompanied by central nervous system (CNS) involvement (6–8). Mild WAS-related manifestations have also been reported in some mothers and other female carriers (9). Without definitive treatment, WAS is associated with substantial morbidity and mortality from bleeding, severe infection, immune dysregulation, and malignancy, particularly in severe early-onset disease (10).

Over the past two decades, outcomes have improved markedly with advances in hematopoietic stem cell transplantation (HSCT) and, more recently, autologous lentiviral gene therapy (11). Contemporary HSCT series report 5-year overall survival rates of approximately 87–91%, and long-term follow-up from gene therapy trials demonstrates sustained correction of key systemic features (12–14). As survival improves, clinical focus has shifted toward long-term complications and post-treatment management in children and young adults with WAS, underscoring the need to recognize uncommon but serious manifestations (15).

Neurological involvement has been described in WAS, but the available evidence is sparse and predominantly case-based, impeding a unified understanding of phenotypes and associated clinical circumstances. We therefore conducted a systematic review to synthesize the published evidence and characterize neurological manifestations in their clinical context.

## 2 Methods

### 2.1 Study identification

Study identification followed the Preferred Reporting Items for Systematic Reviews and Meta-Analyses (PRISMA) guidelines and was pre-registered on PROSPERO (CRD420251141002) (16). A comprehensive search using both MeSH/Emtree subject headings and free-text keywords was conducted across PubMed, Embase, Scopus, and Web of Science on 3 July 2025, with no date restrictions. The strategy combined terms for the target population, neurological involvement, and relevant study designs. Searches were limited to English-language records. Full search queries are provided in **Supplementary Table S1**.

### 2.2 Eligibility criteria

Studies were eligible if they reported at least one central or peripheral neurological manifestation in individuals with WAS, diagnosed by a reported pathogenic or likely pathogenic *WAS* variant, or by a clinician-assigned diagnosis supported by a compatible phenotype. Neurological involvement had to be confirmed by clinical evaluation and/or instrumental diagnostics (e.g., neuroimaging, electrodiagnostic studies, laboratory analyses, or histopathology). The patient-level data had to be sufficient to identify and classify the manifestation. We excluded studies that were non-human or *in vitro*, not in English, lacked full text (e.g., conference abstracts), duplicated other included cases, provided no extractable patient-level neurological data, or did not provide a sufficient diagnostic support for WAS.

### 2.3 Study selection and data extraction

All records were imported into Rayyan, where duplicates were identified and removed. Two reviewers (NGR and VFM) independently screened titles and abstracts, followed by full-text assessment of potentially eligible studies. Discrepancies were resolved by a third reviewer (SG).

Data extraction was performed by the same two reviewers using a predefined form capturing study-level and patient-level variables. Discrepancies were resolved by consensus with the third reviewer. When information was unavailable, it was recorded as not reported (N/R). Extracted study-level variables included first author, year of publication, country of origin, and study design. Patient-level variables included neurological diagnosis, age at WAS diagnosis, age at neurological onset, follow-up duration, clinical outcome (alive or deceased), attributed cause of death, HSCT status, age at HSCT, and viral status when relevant to the neurological diagnosis, such as EBV in CNS lymphoma and John Cunningham virus (JCV) in progressive multifocal leukoencephalopathy (PML).

When multiple neurological events were reported, the earliest was defined as the primary category. Timing relative to HSCT was determined by comparing reported ages at neurological onset and at HSCT; if either was unavailable, it was coded as N/R. Viral status was considered positive when explicitly reported and neurologically relevant; all other cases were coded N/R.

### 2.4 Methodological Appraisal

Methodological quality was assessed according to the study design. Two reviewers (NGR and VFM) independently appraised each included study, with disagreements resolved by a third reviewer (SG). Case reports and individual case letters were evaluated using the Joanna Briggs Institute (JBI) Critical Appraisal Checklist for Case Reports. The single case series was assessed using the JBI Checklist for Case Series, and the cohort study was appraised using the Newcastle–Ottawa Scale (NOS). Appraisal results are presented in **Supplementary Table S2**.

### 2.5 Statistical analyses

Given the rarity of WAS and heterogeneity of reported neurological manifestations, analyses were primarily descriptive and conducted at the patient level using extracted data. Categorical variables were summarized as counts and percentages, and continuous variables as medians with interquartile ranges (IQRs). When data permitted, interval measures were calculated from reported ages. Exploratory comparisons used two-sided Fisher’s exact test for categorical variables and Wilcoxon rank-sum or Kruskal–Wallis tests for continuous variables, interpreted as hypothesis-generating. Missing data were not imputed, and denominators reflect available data for each variable. Statistical analyses were conducted in *R* (version 4.5.1) using the *dplyr*, *tidyr*, *readxl*, and *ggplot2* packages.

## 3 Results

### 3.1 Study selection and characteristics

**Figure 1:**
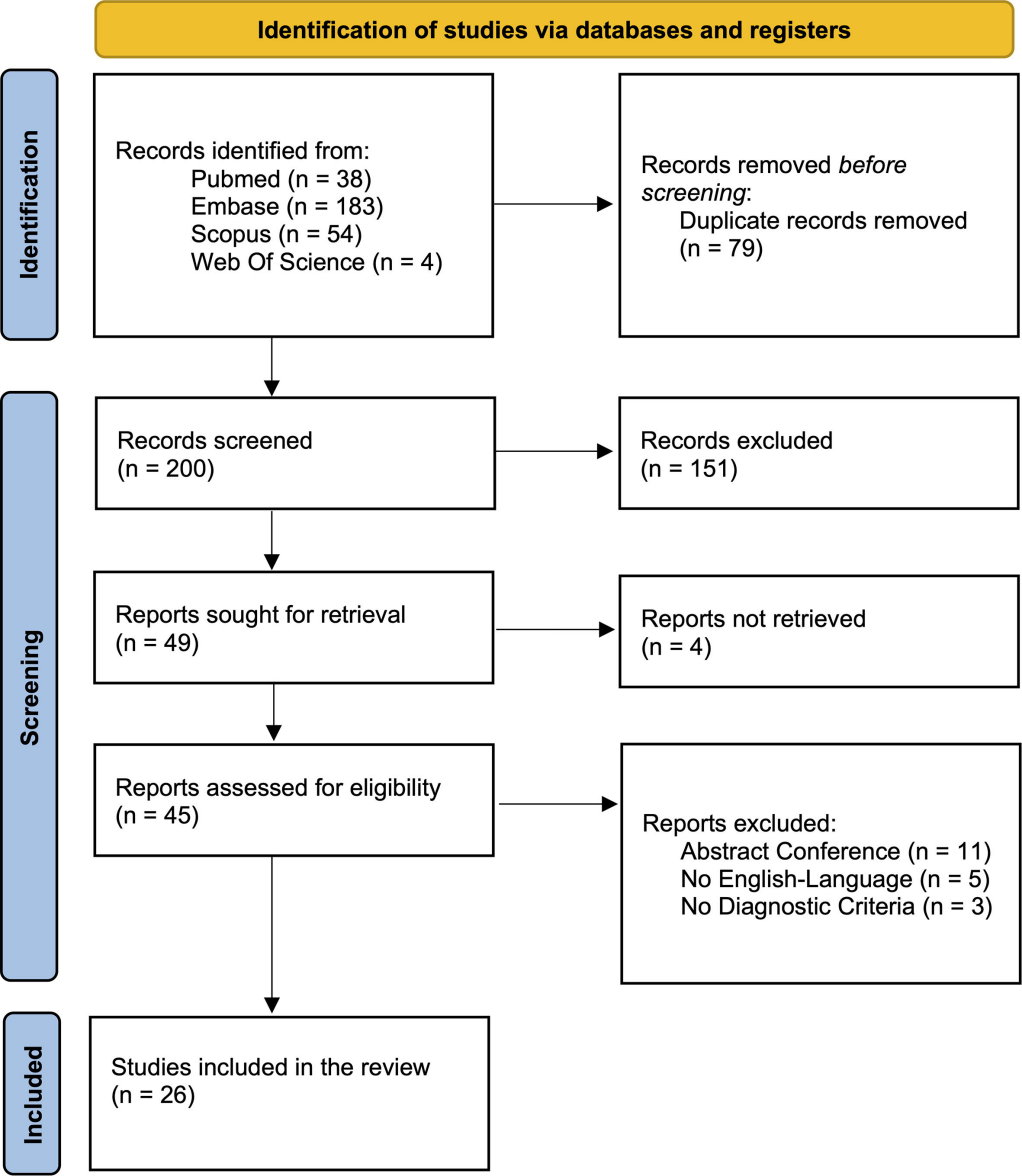
PRISMA flow diagram of study selection.

The initial search yielded 279 records, of which 200 remained after deduplication. After title and abstract screening, 49 reports were sought for full-text assessment. Four full texts could not be retrieved, and 19 were excluded: 11 were conference abstracts without accompanying full text, 5 were non-English publications, and 3 lacked sufficient diagnostic support. Ultimately, 26 studies met the inclusion criteria, collectively describing 32 patients with genetically or clinically confirmed Wiskott–Aldrich syndrome and neurological manifestations (17–42). The study selection process is illustrated in **Figure 1**.

Most included studies were individual case reports or letters (n = 24), alongside one case series and one retrospective cohort study. Publications spanned 1974 to 2025 and mainly originated from Europe, North America, and Asia.

### 3.2 Patient and clinical characteristics

Thirty-two patients with genetically or clinically confirmed WAS and neurological manifestations were identified. Most were pediatric at neurological onset (25/32, 78.1%), whereas 7 (21.9%) had neurological onset in adulthood. The median age at WAS diagnosis was 0.4 years (IQR 0.1–1.0; range 0.0–7.0), and neurological manifestations occurred at a median age of 3.6 years (IQR 2.0–14.5; range 0.0–50.0), corresponding to a within-patient median interval of 3.0 years (IQR 1.1–13.8; range 0.0–49.0) after diagnosis.

HSCT status was available for all patients: 10 (31.2%) had undergone transplantation, and 22 (68.8%) had not. Among the 7 transplanted patients with available timing data, the median interval from WAS diagnosis to HSCT was 1.3 years (IQR 0.9–7.9; range 0.5–15.0); neurological onset occurred before HSCT in 2 patients (28.6%) and after HSCT in 5 (71.4%). In post-HSCT cases, onset occurred shortly after transplantation (median 0.0 years; IQR 0.0–0.08).

The median follow-up after neurological onset was 0.9 years (IQR 0.4–2.5). Of 32 patients, 19 deaths (59.4%; 95% CI 42.3–74.5%) were attributed to the neurological complication in the source reports, and one additional death was unrelated. Neurological event–attributed mortality was 63.6% (14/22; 95% CI 42.9–80.3%) among non-HSCT patients and 50.0% (5/10; 95% CI 23.7–76.3%) among HSCT recipients (p = 0.699). All JCV-positive cases (5/5) were diagnosed with PML and were fatal. Among CNS lymphoma cases with reported EBV status, 3 of 5 were fatal (60.0%; p = 0.089).

Patient-level characteristics are summarized in **Table 1**, and the full extraction table is provided in **Supplementary Table S3**.

**Table 1:** Patient-level clinical features of neurological manifestations in Wiskott–Aldrich syndrome.

ID	Neurological diagnosis	Primary category	WAS onset (years)	Neurological onset (years)	Follow-up (years)	Outcome	Cause of death	HSCT [age at HSCT (years)]	Viral status
P1	Guillain–Barré syndrome (AMSAN variant)	Immune-mediated	0.10	2.00	0.33	Alive	N/R	Yes (1.42)	N/R
P2	Cranial Nerve Palsy (Parapharyngeal Lymphoma)	Neoplastic	0.60	14.00	4.00	Alive	N/R	No	N/R
P3	Bilateral Optic Neuritis (Demyelinating)	Immune-mediated	1.00	25.00	1.00	Alive	N/R	No	N/R
P4	Progressive Multifocal Leukoencephalopathy	Infectious	0.20	14.00	0.20	Deceased	PML-related complications	Yes (4.00)	JCV
P5	CNS Lymphoma (PTLD)	Neoplastic	0.00	2.00	0.16	Deceased	PTLD-related complications	Yes (1.92)	EBV
P6	Intracranial Hemorrhage	Hemorrhagic	0.40	1.40	0.40	Deceased	ICH-related complications	No	N/R
P7	Guillain–Barré syndrome; Hydrocephalus	Immune-mediated	0.40	0.90	2.50	Alive	N/R	Yes (1.00)	N/R
P8	Guillain–Barré syndrome (AIDP variant)	Immune-mediated	0.90	2.60	0.08	Alive	N/R	No	N/R
P9	CNS Lymphoma (B-cell PTLD-like)	Neoplastic	1.0	4.00	1.00	Deceased	PTLD-related complications	No	EBV
P10	CNS Lymphoma (DLBCL non-GCB)	Neoplastic	1.0	22.00	1.00	Deceased	Sepsis-related complications	Yes (N/R)	N/R
P11	CNS Lymphoma (B-cell PTLD-like)	Neoplastic	1.0	2.00	10.00	Alive	N/R	Yes (N/R)	EBV
P12	CNS Lymphoma (B-cell PTLD-like)	Neoplastic	1.0	6.00	2.00	Deceased	PTLD-related complications	No	EBV
P13	CNS Lymphoma (unclassified B-cell LPD)	Neoplastic	1.0	14.00	1.00	Deceased	PTLD-related complications	No	N/R
P14	CNS Lymphoma (B-cell PTLD-like)	Neoplastic	1.0	50.00	5.00	Alive	N/R	No	EBV
P15	Bilateral Optic Neuritis (MOGAD)	Immune-mediated	0.90	5.00	3.00	Alive	N/R	No	N/R
P16	CNS Lymphoma (Reticulum Cell Sarcoma)	Neoplastic	1.00	3.00	0.42	Deceased	Sepsis-related complications	No	N/R
P17	Progressive Multifocal Leukoencephalopathy	Infectious	0.20	14.00	0.16	Deceased	PML-related complications	Yes (14.00)	JCV
P18	Progressive Multifocal Leukoencephalopathy	Infectious	7.00	15.00	0.83	Deceased	PML-related complications	No	JCV
P19	CNS Lymphoma (Diffuse, Perivascular)	Neoplastic	0.20	2.10	0.67	Deceased	ICH-related complications	No	N/R
P20	CNS Lymphoma (High-grade B-cell NHL)	Neoplastic	0.30	3.60	N/R	Deceased	PTLD-related complications	No	N/R
P21	Intraventricular Hemorrhage; Hydrocephalus	Hemorrhagic	0.00	0.0	0.14	Deceased	Unrelated to CNS complication	No	N/R
P22	Intracranial Hemorrhage	Hemorrhagic	0.00	33.00	3.00	Alive	N/R	No	N/R
P23	Intracranial Hemorrhage; Viral Meningitis	Hemorrhagic	0.00	0.0	1.08	Alive	N/R	Yes (0.50)	N/R
P24	Subarachnoid Hemorrhage	Immune-mediated	0.00	15.00	1.00	Alive	N/R	Yes (15.00)	N/R
P25	Intraparenchymal Hemorrhage; Cerebral Atrophy	Hemorrhagic	2.00	2.00	0.0	Deceased	ICH-related complications	No	N/R
P26	Amoebic Encephalitis	Infectious	0.90	2.00	0.04	Deceased	Encephalitis-related complications	Yes (2.00)	N/R
P27	Progressive Multifocal Leukoencephalopathy	Infectious	0.10	36.00	0.40	Deceased	PML-related complications	No	JCV
P28	Progressive Multifocal Leukoencephalopathy	Infectious	N/R	20.00	0.33	Deceased	PML-related complications	No	JCV
P29	Intracranial Hemorrhage; Hydrocephalus	Hemorrhagic	0.00	1.10	1.30	Deceased	ICH-related complications	No	N/R
P30	Intracranial Hemorrhage; Hydrocephalus; Meningitis	Hemorrhagic	1.00	1.20	N/R	Alive	N/R	No	N/R
P31	CNS Lymphoma (Reticulum Cell Sarcoma)	Neoplastic	0.10	19.00	0.38	Deceased	Sepsis-related complications	No	N/R
P32	Intracranial Hemorrhage	Hemorrhagic	0.00	0.00	1.00	Deceased	ICH-related complications	No	N/R

CNS, central nervous system; DLBCL, diffuse large B-cell lymphoma; EBV, Epstein–Barr virus; GCB, germinal center B-cell; HSCT, hematopoietic stem cell transplantation; ID, patient identification; JCV, John Cunningham virus; LPD, lymphoproliferative disease; MOGAD, myelin oligodendrocyte glycoprotein antibody–associated disease; NHL, non-Hodgkin lymphoma; PTLD, post-transplant lymphoproliferative disorder.

### 3.3 Neurological involvement

**Figure 2:**
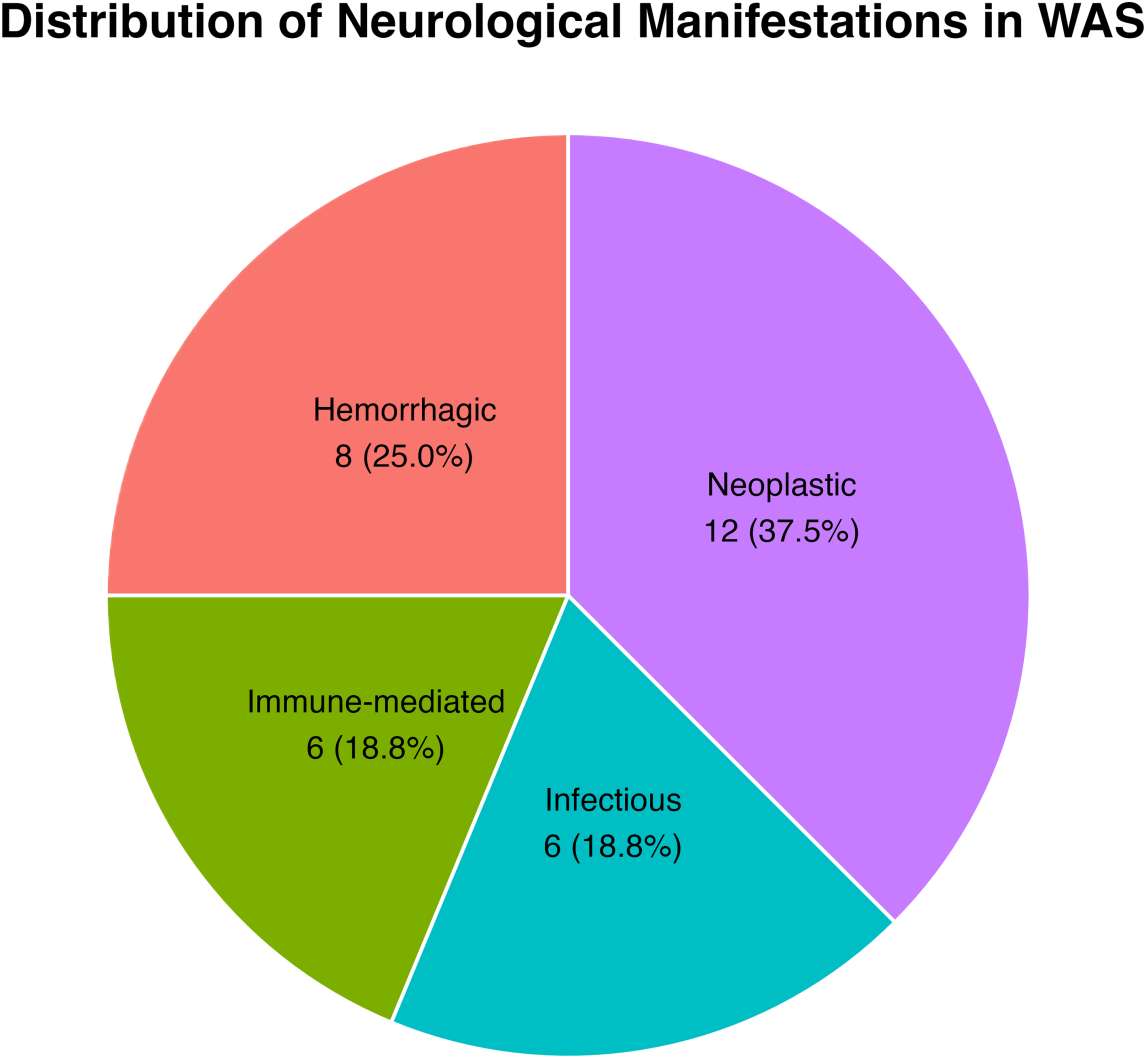
Distribution of primary neurological categories in Wiskott–Aldrich syndrome. Pie chart showing the proportion of WAS patients in each primary neurological phenotype.

Neurological diagnoses were classified into four categories: hemorrhagic (n = 8), immune-mediated (n = 6), infectious (n = 6), and neoplastic (n = 12). **Figure 2** illustrates their distribution. **Table 2** summarizes category-specific age at onset, mortality, and HSCT status.

**Table 2:** Summary of neurological involvement in patients with Wiskott–Aldrich syndrome.

Category	N (%)	Median age at onset, years (IQR; range)	Number of deaths (%)	HSCT (Before/After/N/R)
Hemorrhagic	8 (25.0%)	1.15 (0.00–1.55; 0–33)	5 (62.5%)	7/1/0
Immune-mediated	6 (18.8%)	3.80 (2.15–12.50; 0.9–25)	0 (0%)	3/3/0
Infectious	6 (18.8%)	14.50 (14.00–18.75; 2–36)	6 (100%)	3/2/1
Neoplastic	12 (37.5%)	5.00 (2.77–15.25; 2–50)	9 (75.0%)	9/1/2

HSCT, hematopoietic stem cell transplantation; IQR, interquartile range.

Brain hemorrhagic events were observed in 8 patients (25.0%), spanning intracranial, intraventricular, and subarachnoid hemorrhage. The median age at neurological onset was 1.15 years (IQR 0.00–1.55; range 0–33). In half of the cases (4/8), hemorrhage occurred before or concurrently with WAS diagnosis. Deaths occurred in 5 cases (62.5%), although one was not attributed to the neurological event. Neurological onset preceded HSCT in 7 patients and followed HSCT in 1.

Immune-mediated events occurred in 6 patients (18.8%), including Guillain–Barré syndrome (GBS) variants and optic neuritis, including one case of myelin oligodendrocyte glycoprotein antibody–associated disease (MOGAD). The median age at onset was 3.80 years (IQR 2.15–12.50; range 0.9–25). No deaths were reported in this subgroup. Neurological onset preceded HSCT in 3 cases and followed HSCT in 3.

Infectious events were identified in 6 patients (18.8%), comprising five cases of progressive multifocal leukoencephalopathy (PML) and one case of bacterial meningitis. The median age at onset was 14.50 years (IQR 14.00–18.75; range 2–36). All patients died (6/6). All five PML cases (15.6% of 32 WAS patients) were JCV-positive. Neurological onset preceded HSCT in 3 cases and followed HSCT in 2.

Neoplastic manifestations affected 12 patients (37.5%) and predominantly included CNS lymphoma and reticulum cell sarcoma (historical terminology). The median age at onset was 5.00 years (IQR 2.77–15.25; range 2–50). Fatality was 75.0% (9/12). Neurological onset preceded HSCT in 9 cases and followed HSCT in 1; the timing relative to HSCT was unreported in 2 cases. EBV positivity was reported in 5 cases.

**Figure 3:**
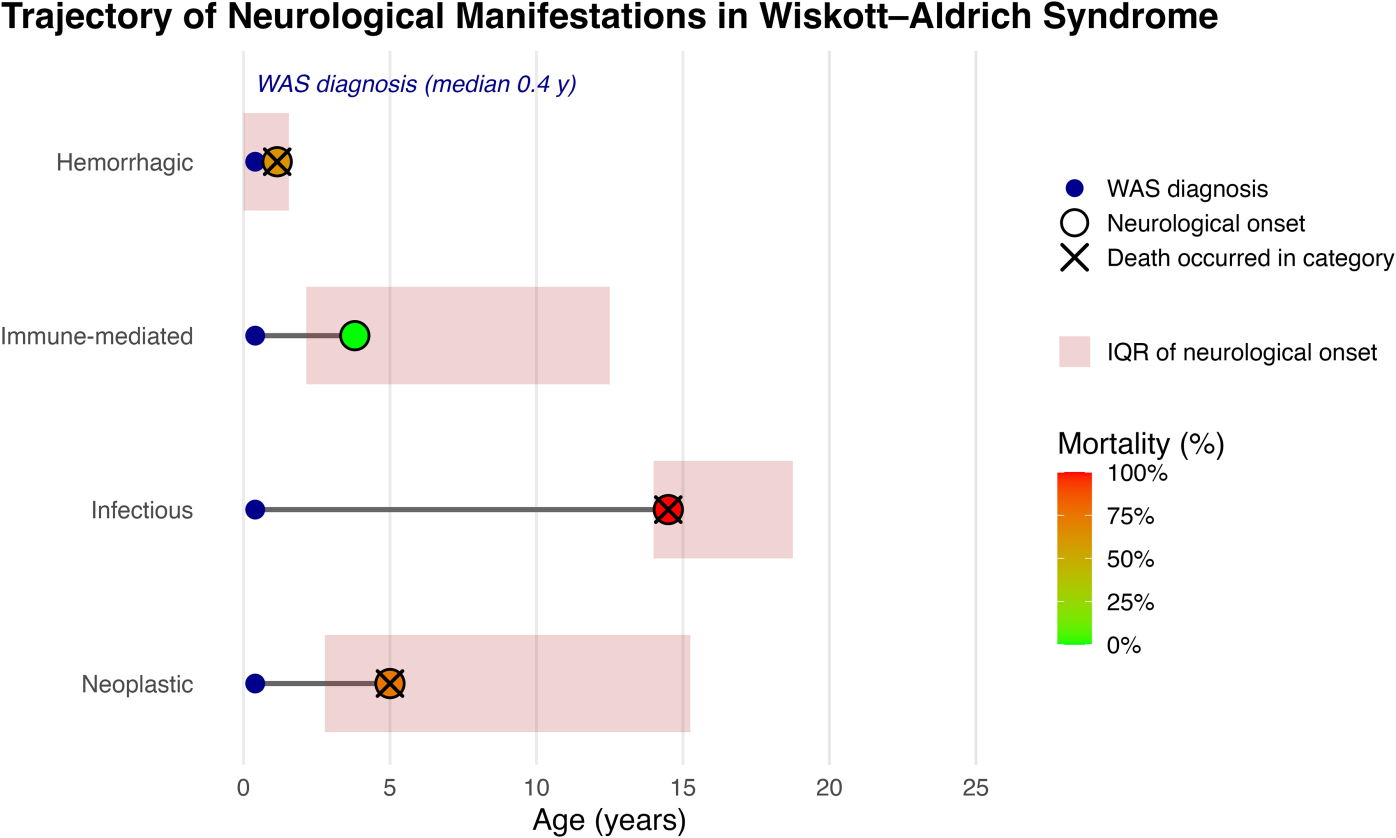
Trajectory of neurological complications in Wiskott–Aldrich syndrome. Blue circles indicate WAS diagnosis (median 0.4 years); colored circles denote neurological onset by phenotype, with interquartile ranges shaded in red. Color intensity reflects phenotype-specific mortality. WAS, Wiskott–Aldrich syndrome; IQR, interquartile range.

Age at neurological onset differed significantly across categories (χ² = 10.07, df = 3, p = 0.018), as did case-fatality (p = 0.002). These comparisons are illustrated in **Figure 3**.

## 4 Discussion

This systematic review synthesizes 32 published cases describing neurological involvement in Wiskott–Aldrich syndrome and therefore reflects reported manifestations within a rare disease rather than population-level risk or incidence. Owing to the exclusively case-based nature of the evidence and the absence of a defined denominator, the findings should be interpreted as patterns observed among reported cases rather than estimates of frequency or comparative risk. Within these constraints, age at neurological onset differed across categories (p = 0.018), and case-fatality varied by phenotype (p = 0.002). Neurological events were reported both before and after HSCT. Although case-fatality appeared higher in non-HSCT than HSCT patients, this comparison is underpowered and should not be interpreted as evidence of a treatment effect.

Brain hemorrhagic events were reported at the youngest ages (median 1.15 years) and preceded WAS diagnosis in half of the cases, consistent with early bleeding vulnerability related to congenital thrombocytopenia. Immune-mediated manifestations were reported in childhood (median 3.8 years) and ranged from GBS variants and optic neuritis to MOGAD, with the latter emerging as a potentially underrecognized phenotype. These events were observed both before and after HSCT and were non-fatal in available cases. Infectious neurological manifestations were reported at older ages (median 14.5 years) and dominated by JCV-positive PML, which was uniformly fatal in reported cases, plausibly in the setting of advanced immune dysfunction and, in some patients, antecedent immunosuppression. These observations align with recent literature suggesting that PML may be a severe, potentially underrecognized opportunistic neurological complication in WAS and other IEI (43, 44). Neoplastic involvement was the most reported category, spanned a wide age range (median 5.0 years), and predominantly consisted of CNS lymphoma, with high case-fatality and EBV positivity in a subset of tested cases.

Neurological involvement in WAS is consistent with patterns observed across IEI, where CNS manifestations often reflect immune dysregulation, impaired pathogen control, or treatment-related immunosuppression (45, 46). Rather than indicating primary neurological pathology, the phenotypes observed in this review likely reflect systemic mechanisms intrinsic to WAS. These include hemorrhage linked to severe microthrombocytopenia, autoimmune phenomena driven by immune dysregulation, and opportunistic infections or EBV-driven neoplasia arising in the setting of profound or iatrogenically augmented immunodeficiency. Experimental data further support a neuroimmune role for WASp. In murine models, WASP deficiency attenuates CNS autoimmunity through impaired CNS T-cell trafficking, reduced CNS inflammation and demyelination, and defective microglial activation (47). Complementary *in vitro* studies also show altered microglial inflammatory signaling and reduced neurotoxicity in the absence of normal WASP function (48). These findings support a plausible link between WAS-associated immune dysregulation and CNS vulnerability, while indicating that current models are better interpreted as mechanistic rather than phenotypic correlates of human neurological disease

The observed patterns have practical implications for interpreting neurological symptoms across the WAS lifespan. Differences in age at onset and case-fatality across reported manifestations support context- and phenotype-informed triage, with a low threshold for urgent evaluation of infantile hemorrhagic events and late-onset infectious or neoplastic presentations. **Table 3** outlines age-stratified clinical considerations to support recognition and triage (49–52). Multidisciplinary coordination among hematology, immunology, and neurology can facilitate prompt neuroimaging and targeted viral testing when clinically indicated, particularly after HSCT or in the setting of significant immunosuppression. Families and primary physicians should be educated on neurological red flags, and a standardized Emergency Department letter may expedite evaluation in urgent-care settings (53). Earlier recognition and timely escalation may reduce diagnostic delays and prevent avoidable morbidity (54).

**Table 3:** Age-stratified clinical considerations for neurological complications in WAS, synthesized from this review to support recognition and triage (not to prescribe surveillance).

Age group	Neurological manifestation	Key clinical triggers	Diagnostic workup	Management considerations
Infancy (0–2 years)	Hemorrhagic (e.g., ICH, IVH, SAH)	Seizures, bulging fontanelle, vomiting, neurologic decline, petechiae, or other bleeding signs; relevant family history	Urgent neuroimaging (CT/MRI), platelet count, coagulation profile, genetic testing for WAS if clinically indicated	Immediate stabilization; baseline neurodevelopmental assessment; repeat imaging and labs as needed; consider structured neurodevelopmental follow-up
Early to Middle Childhood (2–10 years)	Immune-mediated (e.g., GBS, optic neuritis, MOGAD)	Progressive weakness, ataxia, areflexia, visual disturbances	MRI brain and spine, CSF analysis if demyelination suspected, serum autoantibody panel (e.g., MOG, AQP4, ANA)	Immunomodulatory therapy (e.g., IVIG, corticosteroids); periodic neurological evaluation, especially following immune-mediated events
Late Childhood to Adolescence (>10 years, post-HSCT)	Opportunistic infections and neoplasms (e.g., PML, CNS lymphoma)	Cognitive decline, focal deficits, seizures, visual changes	Brain MRI; CSF analysis with viral PCR when indicated (e.g., JCV); EBV monitoring per institutional HSCT protocols	Maintain low threshold for neuroimaging and viral testing; ensure integration within transplant/immunology follow-up; individualized surveillance as clinically appropriate

ANA, antinuclear antibody; AQP4, aquaporin-4; CSF, cerebrospinal fluid; CT, computed tomography; EBV, Epstein–Barr virus; GBS, Guillain–Barré syndrome; HSCT, hematopoietic stem cell transplantation; ICH, intracranial hemorrhage; IVIG, intravenous immunoglobulin; IVH, intraventricular hemorrhage; JCV, John Cunningham virus; MOG, myelin oligodendrocyte glycoprotein; MOGAD, myelin oligodendrocyte glycoprotein antibody-associated disease; MRI, magnetic resonance imaging; PCR, polymerase chain reaction; PML, progressive multifocal leukoencephalopathy; SAH, subarachnoid hemorrhage; WAS, Wiskott-Aldrich Syndrome.

Advancing care for WAS will require stronger data infrastructure, deeper mechanistic insight, and continued progress in curative approaches. Future registries and cohorts should incorporate prespecified neurological phenotyping and harmonized documentation of HSCT timing and exposures, immunosuppression history, and viral diagnostics (e.g., EBV and JCV testing) to enhance outcome characterization, identify candidate risk modifiers (e.g., genotype, immune reconstitution), and inform symptom-triggered triage in routine care (55, 56). Mechanistic studies are also needed to clarify how microthrombocytopenia, immune dysregulation, and impaired antiviral surveillance contribute to CNS vulnerability. In our review, neurological manifestations were reported more often after than before HSCT (5 vs. 2 among patients with timing data), suggesting a potential peri-transplant window of vulnerability. Whether gene therapy with etuvetidigene autotemcel (Waskyra) reduces neurological risk remains unknown and warrants prospective evaluation following its 2025 regulatory approvals in Europe and the United States for patients lacking an HLA-matched related donor (57, 58).

This review is limited by the rarity of neurological involvement in WAS and the predominance of case-based literature. Although the search was systematic, most included records were case reports or letters, increasing the risk of publication and selection bias toward atypical or severe presentations. Consequently, observed patterns of phenotype distribution, age at onset, and case-fatality should not be interpreted as population-level estimates. The absence of a defined denominator precludes estimation of incidence or prevalence. Case identification likely varied by diagnostic era, intensity, and setting (e.g., availability of magnetic resonance imaging, cerebrospinal fluid analysis, and viral testing), introducing time- and system-dependent ascertainment bias. Reporting was frequently incomplete for key variables (e.g., HSCT timing and exposures, diagnostic strategy, antecedent immunosuppression, long-term follow-up), limiting assessment of effect modifiers and precluding causal inference. While JBI and NOS appraisal scores were generally moderate to high, recurrent gaps in timelines, follow-up, and diagnostic detail constrain the strength and generalizability of conclusions. Nonetheless, the review was PRISMA-aligned, PROSPERO-registered, and applied validated appraisal tools, providing the first systematic synthesis of neurological manifestations in WAS.

## 5 Conclusion

Among 32 reported patients with WAS and neurological involvement, phenotypes differed in age at onset and case-fatality. Hemorrhagic events occurred earliest and often preceded or coincided with WAS diagnosis; immune-mediated manifestations arose in childhood and were non-fatal in available cases; and infectious and neoplastic phenotypes carried high mortality. Notably, 5 patients developed fatal JCV-associated PML, suggesting that this may be an under-recognized neurological manifestation of WAS. Given the case-derived nature of the evidence and the absence of a defined denominator, these findings should be interpreted as descriptive patterns rather than estimates of comparative risk. Nonetheless, they support heightened clinical vigilance across the disease course, including both pre- and post-HSCT periods.

## Data Availability

The original contributions presented in the study are included in the article/**Supplementary Material**. Further inquiries can be directed to the corresponding author.
