# Supplementary material for: Neurological manifestations in Wiskott–Aldrich syndrome: a systematic review
Source: Front Immunol. 2026 May 7;17:1829058. doi: 10.3389/fimmu.2026.1829058 (PMC13189806; doi:10.3389/fimmu.2026.1829058)
Supplement: Supplementary file 1 [file Table1.docx]

**Supplementary Materials**

# Supplementary Table S1. Search Strategy

Complete search strings used for PubMed, Embase, Scopus, and Web of Science (queried on July 3, 2025), with no restrictions on language or publication date. Study design filters were applied when available.

| **Database** | **Platform** | **Date** | **Search String** | **Results** |
| --- | --- | --- | --- | --- |
| PubMed | NCBI | 03/07/2025 | ("Wiskott-Aldrich Syndrome"[Mesh] OR "Wiskott-Aldrich Syndrome"[tiab] OR "Wiskott Aldrich Syndrome"[tiab]) AND ( "Nervous System Diseases"[Mesh] OR "Neurologic Manifestations"[tiab] OR "central nervous system"[tiab] OR “neurologic complications”[tiab] OR "CNS"[tiab] OR "seizures"[Mesh] OR "epilepsy"[tiab] OR "intracranial hemorrhage"[tiab] OR "cerebral hemorrhage"[tiab] OR "intraventricular hemorrhage"[tiab] OR "cognitive impairment"[tiab] OR "neurodevelopmental delay"[tiab] OR "encephalopathy"[tiab] OR "brain tumor"[tiab] OR "CNS lymphoma"[tiab] OR "progressive multifocal leukoencephalopathy"[tiab] OR "Guillain-Barre syndrome"[Mesh] OR "autoimmune neuropathy"[tiab] OR "cerebrovascular"[tiab] OR "stroke"[tiab] OR "hydrocephalus"[tiab] OR "maculopathy"[tiab] OR "vestibular failure"[tiab] OR "deafness"[tiab] ) AND ( "case reports"[Publication Type] OR "case series"[tiab] OR "observational study"[Publication Type] OR "cohort study"[tiab]) | 38 |
| Embase | Elsevier | 03/07/2025 | ('wiskott aldrich syndrome'/exp OR 'wiskott aldrich syndrome':ti,ab) AND ('nervous system disease'/exp OR 'neurologic manifestation':ti,ab OR 'neurologic complications':ti,ab OR 'central nervous system':ti,ab OR 'cns':ti,ab OR 'seizure'/exp OR 'epilepsy':ti,ab OR 'intracranial hemorrhage':ti,ab OR 'cerebral hemorrhage':ti,ab OR 'intraventricular hemorrhage':ti,ab OR 'cognitive impairment':ti,ab OR 'neurodevelopmental delay':ti,ab OR 'encephalopathy':ti,ab OR 'brain tumor':ti,ab OR 'cns lymphoma':ti,ab OR 'progressive multifocal leukoencephalopathy':ti,ab OR 'guillain barre syndrome'/exp OR 'autoimmune neuropathy':ti,ab OR 'cerebrovascular disorder':ti,ab OR 'stroke':ti,ab OR 'hydrocephalus':ti,ab OR 'maculopathy':ti,ab OR 'vestibular failure':ti,ab OR 'deafness':ti,ab) AND ('case report'/exp OR 'case series':ti,ab OR 'observational study'/exp OR 'cohort analysis'/exp OR 'cohort study':ti,ab) | 183 |
| Scopus | Elsevier | 03/07/2025 | TITLE-ABS-KEY ( "Wiskott-Aldrich Syndrome" OR "Wiskott Aldrich Syndrome" ) AND TITLE-ABS-KEY ( "Nervous System Diseases" OR "Neurologic Manifestations" OR "neurologic complications" OR "central nervous system" OR "CNS" OR "seizures" OR "epilepsy" OR "intracranial hemorrhage" OR "cerebral hemorrhage" OR "intraventricular hemorrhage" OR "cognitive impairment" OR "neurodevelopmental delay" OR "encephalopathy" OR "brain tumor" OR "CNS lymphoma" OR "progressive multifocal leukoencephalopathy" OR "Guillain-Barre syndrome" OR "autoimmune neuropathy" OR "cerebrovascular" OR "stroke" OR "hydrocephalus" OR "maculopathy" OR "vestibular failure" OR "deafness") AND TITLE-ABS-KEY ( "case report" OR "case series" OR "observational study" OR "cohort study") | 54 |
| Web of Science | Clarivate | 03/07/2025 | TS=("Wiskott-Aldrich Syndrome" OR "Wiskott Aldrich Syndrome") AND TS=("Nervous System Diseases" OR "Neurologic Complications" OR"Neurologic Manifestations" OR "central nervous system" OR CNS OR seizures OR epilepsy OR "intracranial hemorrhage" OR "cerebral hemorrhage" OR "intraventricular hemorrhage" OR "cognitive impairment" OR "neurodevelopmental delay" OR encephalopathy OR "brain tumor" OR "CNS lymphoma" OR "progressive multifocal leukoencephalopathy" OR "Guillain-Barre syndrome" OR "autoimmune neuropathy" OR cerebrovascular OR stroke OR hydrocephalus OR maculopathy OR "vestibular failure" OR deafness) AND TS=("case report" OR "case series" OR "observational study" OR "cohort study") | 4 |

# Supplementary Table S2. Methodological Appraisal

Methodological quality appraisal of included studies using validated tools: the Joanna Briggs Institute (JBI) checklists for case reports, letters, and case series, and the Newcastle–Ottawa Scale (NOS) for the cohort study.

a) Case Reports / Case Letters (JBI Checklist)

| Author, Year | Patient ID | JBI Items (1–8) | Score (0–8) | Overall RoB |
| --- | --- | --- | --- | --- |
| Broomall et al., 2017 [1]. | P1 | yes, no, yes, yes, yes, yes, no, yes | 6 | Moderate |
| Rampisela et al., 2010 [2]. | P2 | yes, no, yes, yes, no, yes, no, yes | 5 | Moderate |
| Moschos et al., 1999 [3]. | P3 | yes, no, yes, yes, yes, yes, no, yes | 6 | Moderate |
| Yasuda et al., 2003 [4]. | P4 | yes, yes, yes, yes, yes, yes, no, yes | 7 | Low |
| Wang et al., 2005 [5]. | P5 | yes, yes, yes, yes, yes, yes, no, yes | 7 | Low |
| Yoshida et al., 1997 [6]. | P6 | yes, yes, yes, yes, no, yes, no, yes | 6 | Moderate |
| Ávila-Smirnow et al., 2020 [7]. | P7 | yes, yes, yes, yes, yes, yes, yes, yes | 8 | Low |
| Ölmez et al., 2008 [8]. | P8 | yes, yes, yes, yes, yes, yes, no, yes | 7 | Low |
| Xie et al., 2023 [9]. | P15 | yes, yes, yes, yes, yes, yes, yes, yes | 8 | Low |
| Model et al., 1977 [10]. | P16 | yes, yes, yes, yes, yes, yes, yes, yes | 8 | Low |
| Yasuda et al., 2008 [11]. | P17 | yes, yes, yes, yes, yes, yes, no, yes | 7 | Low |
| Katz et al., 1994.[12] | P18 | yes, yes, yes, yes, no, yes, yes, yes | 7 | Low |
| Hutter et al., 1981 [13]. | P19 | yes, yes, yes, yes, yes, yes, no, yes | 7 | Low |
| Vignesh et al., 2017 [14]. | P20 | yes, yes, yes, yes, no, no, no, yes | 5 | Moderate |
| Sero et al., 2022 [15]. | P21 | yes, yes, yes, yes, yes, yes, no, yes | 7 | Low |
| Gaspoz et. Al, 1995 [16]. | P22 | yes, yes, yes, yes, yes, yes, no, yes | 7 | Low |
| Kreetapirom et al., 2017 [17]. | P23 | yes, yes, yes, yes, yes, yes, yes, yes | 8 | Low |
| Conyers et al., 2016 [18]. | P24 | yes, yes, yes, yes, yes, yes, yes, yes | 8 | Low |
| Rai et al., 2025 [19]. | P25 | yes, yes, yes, yes, yes, yes, no, yes | 7 | Low |
| Ahuja et al., 2023 [20]. | P26 | yes, yes, yes, yes, yes, yes, no, yes | 7 | Low |
| Matsushima et al., 1997 [21]. | P27 | yes, yes, yes, yes, yes, yes, no, yes | 7 | Low |
| Downes et al., 2001 [22]. | P28 | yes, yes, yes, yes, no, yes, no, yes | 6 | Moderate |
| Heidelberger et al., 1974 [23]. | P31 | yes, yes, yes, yes, yes, yes, yes, yes | 8 | Low |
| Sun et al., 2024 [24] . | P32 | yes, yes, yes, yes, yes, yes, no, yes | 7 | Low |

b) Case Series (JBI Checklist)

| Author, Year | Patient ID | JBI Items (1–10) | Score (0–10) | Overall RoB |
| --- | --- | --- | --- | --- |
| Harfi et al., 1992 [25]. | P29, P30 | yes, yes, yes, no, no, yes, yes, yes, no, no | 6 | Moderate |

c) Cohort Studies (Newcastle–Ottawa Scale, NOS)

| Author, Year | Patient ID | Selection (0–4) | Comparability (0–2) | Outcome (0–3) | Total Stars (0–9) | Overall RoB |
| --- | --- | --- | --- | --- | --- | --- |
| Cheminant et al., 2019 [26]. | P09, P10, P11, P12, P13, P14 | 4 | 0 | 2 | 6 | Moderate |

# Supplementary Table S3. Full Data Extraction Table

Study- and patient-level data extracted from included reports of Wiskott–Aldrich Syndrome with neurological involvement. Variables include study characteristics (author, year, country, design) and patient-level details (neurological diagnosis, age at WAS and symptom onset, outcome, HSCT status and timing, viral status, and follow-up).

| Author, year | Country | Study Design | ID | Neurological Diagnosis | Primary Category | WAS Onset (years) | Neurological Onset (years) | Follow-up (years) | Outcome | Mortality Cause | HSCT (years) | Viral State |
| --- | --- | --- | --- | --- | --- | --- | --- | --- | --- | --- | --- | --- |
| Broomall et al., 2017 [1]. | USA | Case report | P01 | Guillain-Barré Syndrome (AMSAN variant) | Immune-mediated | 0.10 | 2.00 | 0.33 | Alive | N/R | Yes (1.42) | N/R |
| Rampisela et al., 2010 [2]. | USA | Case report | P02 | Cranial Nerve Palsy (Parapharyngeal Lymphoma) | Neoplastic | 0.60 | 14.00 | 4.00 | Alive | N/R | No | N/R |
| Moschos et al., 1999 [3]. | Greece | Case report | P03 | Bilateral Optic Neuritis (Demyelinating) | Immune-mediated | 1.00 | 25.00 | 1.00 | Alive | N/R | No | N/R |
| Yasuda et al., 2003 [4]. | Japan | Case report | P04 | Progressive Multifocal Leukoencephalopathy (PML) | Infectious | 0.20 | 14.00 | 0.20 | Deceased | PML-related complications | Yes (4.00) | JCV |
| Wang et al., 2005 [5]. | Taiwan | Case report | P05 | CNS Lymphoma (PTLD) | Neoplastic | 0.00 | 2.00 | 0.16 | Deceased | PTLD-related complications | Yes (1.92) | EBV |
| Yoshida et al., 1997 [6]. | Japan | Case report | P06 | Intracranial Hemorrhage (ICH) | Hemorrhagic | 0.40 | 1.40 | 0.40 | Deceased | ICH-related complications | No | N/R |
| Ávila-Smirnow et al., 2020 [7]. | Chile | Case report | P07 | Guillain-Barré Syndrome; Hydrocephalus | Immune-mediated | 0.40 | 0.90 | 2.50 | Alive | N/R | Yes (1.00) | N/R |
| Ölmez et al., 2008 [8]. | Turkey | Case letter | P08 | Guillain-Barré Syndrome (AIDP variant) | Immune-mediated | 0.90 | 2.60 | 0.08 | Alive | N/R | No | N/R |
| Cheminant et al., 2019 [26]. | France | Retrospective cohort study (6/13 patients) | P09 | CNS Lymphoma (B-cell PTLD-like) | Neoplastic | 1.0 (0-3.0) | 4.00 | 1.00 | Deceased | PTLD-related complications | No | EBV |
|  |  |  | P10 | CNS Lymphoma (DLBCL non-GCB) | Neoplastic | 1.0 (0-3.0) | 22.00 | 1.00 | Deceased | Sepsis-related complications | Yes (N/R) | N/R |
|  |  |  | P11 | CNS Lymphoma (B-cell PTLD-like) | Neoplastic | 1.0 (0-3.0) | 2.00 | 10.00 | Alive | N/R | Yes (N/R) | EBV |
|  |  |  | P12 | CNS Lymphoma (B-cell PTLD-like) | Neoplastic | 1.0 (0-3.0) | 6.00 | 2.00 | Deceased | PTLD-related complications | No | EBV |
|  |  |  | P13 | CNS Lymphoma (unclassified B-cell LPD) | Neoplastic | 1.0 (0-3.0) | 14.00 | 1.00 | Deceased | PTLD-related complications | No | N/R |
|  |  |  | P14 | CNS Lymphoma (B-cell PTLD-like) | Neoplastic | 1.0 (0-3.0) | 50.00 | 5.00 | Alive | N/R | No | EBV |
| Xie et al., 2023 [9]. | USA | Case report | P15 | Bilateral Optic Neuritis (MOGAD) | Immune-mediated | 0.90 | 5.00 | 3.00 | Alive | N/R | No | N/R |
| Model et al., 1977 [10]. | USA | Case report | P16 | CNS Lymphoma (Reticulum Cell Sarcoma) | Neoplastic | 1.00 | 3.00 | 0.42 | Deceased | Sepsis-related complications | No | N/R |
| Yasuda et al., 2008 [11]. | Japan | Case report | P17 | Progressive Multifocal Leukoencephalopathy (PML) | Infectious | 0.20 | 14.00 | 0.16 | Deceased | PML-related complications | Yes (14.00) | JCV |
| Katz et al., 1994.[12] | USA | Case report | P18 | Progressive Multifocal Leukoencephalopathy (PML) | Infectious | 7.00 | 15.00 | 0.83 | Deceased | PML-related complications | No | JCV |
| Hutter et al., 1981 [13]. | USA | Case report | P19 | CNS Lymphoma (Diffuse, Perivascular) | Neoplastic | 0.20 | 2.10 | 0.67 | Deceased | ICH-related complications | No | N/R |
| Vignesh et al., 2017 [14]. | India | Case report | P20 | CNS Lymphoma (High-grade B-cell NHL) | Neoplastic | 0.30 | 3.60 | N/R | Deceased | PTLD-related complications | No | N/R |
| Sero et al., 2022 [15]. | Turkey | Case report | P21 | Intraventricular Hemorrhage (IVH); Hydrocephalus | Hemorrhagic | 0.00 | 0.0 | 0.14 | Deceased | Unrelated to CNS complication | No | N/R |
| Gaspoz et. Al, 1995 [16]. | Switzerland | Case report | P22 | Intracranial Hemorrhage (ICH) | Hemorrhagic | 0.00 | 33.00 | 3.00 | Alive | N/R | No | N/R |
| Kreetapirom et al., 2017 [17]. | Thailand | Case letter | P23 | Intracranial Hemorrhage (ICH); Viral Meningitis | Hemorrhagic | 0.00 | 0.0 | 1.08 | Alive | N/R | Yes (0.50) | N/R |
| Conyers et al., 2016 [18]. | Australia | Case letter | P24 | Subarachnoid Hemorrhage (SAH); (Systemic Vaculitis) | Immune-mediated | 0.00 | 15.00 | 1.00 | Alive | N/R | Yes (15.00) | N/R |
| Rai et al., 2025 [19]. | India | Case report | P25 | Intraparenchymal Hemorrhage; Cerebral Atrophy | Hemorrhagic | 2.00 | 2.00 | 0.0 | Deceased | ICH-related complications | No | N/R |
| Ahuja et al., 2023 [20]. | India | Case letter | P26 | Amoebic Encephalitis | Infectious | 0.90 | 2.00 | 0.04 | Deceased | Encephalitis-related complications | Yes (2.00) | N/R |
| Matsushima et al., 1997 [21]. | Japan | Case report | P27 | Progressive Multifocal Leukoencephalopathy (PML) | Infectious | 0.10 | 36.00 | 0.40 | Deceased | PML-related complications | No | JCV |
| Downes et al., 2001 [22]. | UK | Case report | P28 | Progressive Multifocal Leukoencephalopathy (PML) | Infectious | N/R | 20.00 | 0.33 | Deceased | PML-related complications | No | JCV |
| Harfi et al., 1992 [25]. | Saudi Arabia | Case series (2/5 patients) | P29 | Intracranial Hemorrhage (ICH); Hydrocephalus | Hemorrhagic | 0.00 | 1.10 | 1.30 | Deceased | ICH-related complications | No | N/R |
|  |  |  | P30 | Intracranial Hemorrhage (ICH); Hydrocephalus; Meningitis | Hemorrhagic | 1.00 | 1.20 | N/R | Alive | N/R | No | N/R |
| Heidelberger et al., 1974 [23]. | USA | Case report | P31 | CNS Lymphoma (Reticulum Cell Sarcoma) | Neoplastic | 0.10 | 19.00 | 0.38 | Deceased | Sepsis-related complications | No | N/R |
| Sun et al., 2024 [24] . | China | Case letter | P32 | Intracranial Hemorrhage (ICH) | Hemorrhagic | 0.00 | 0.00 | 1.00 | Deceased | ICH-related complications | No | N/R |

*Abbreviations: ID, Patient Identification; AMSAN, acute motor–sensory axonal neuropathy; AIDP, acute inflammatory demyelinating polyneuropathy; PML, progressive multifocal leukoencephalopathy; PTLD, post-transplant lymphoproliferative disorder; LPD, lymphoproliferative disease; DLBCL, diffuse large B-cell lymphoma; GCB, germinal center B-cell; MOGAD, myelin oligodendrocyte glycoprotein antibody–associated disease; ICH, intracranial hemorrhage; IVH, intraventricular hemorrhage; SAH, subarachnoid hemorrhage; CNS, central nervous system; HSCT, hematopoietic stem cell transplantation; JCV, JC virus, EBV, Epstein-Barr virus.*

# Included Studies

Studies included in the systematic review and referenced in Supplementary Tables S2 and S3.

[1] Broomall E, Taylor JM, Peariso K. A 2-year-old boy with difficulty waking after bone marrow transplantation. Seminars in Pediatric Neurology 2018;26:120–3. https://doi.org/10.1016/j.spen.2017.03.018.

[2] Rampisela D, Donner LR. An unusual self-limited clonal Mott cell proliferation with lymphoplasmacytic lymphoma-like features in a child with the Wiskott-Aldrich syndrome and Von Recklinghausen’s neurofibromatosis. Pathol Res Pract 2010;206:467–71. https://doi.org/10.1016/j.prp.2009.07.003.

[3] Moschos M, Parikakis S. Bilateral optic atrophy in a case of Wiskott-Aldrich syndrome. Ann Ophthalmol Glaucoma 1999;31:91–3.

[4] Yasuda Y, Yabe H, Inoue H, Shimizu T, Yabe M, Yogo Y, et al. Comparison of PCR-amplified JC virus control region sequences from multiple brain regions in PML. Neurology 2003;61:1617–9. https://doi.org/10.1212/01.WNL.0000096147.47128.7D.

[5] Wang IJ, Lu MY, Chiang BL, Lin WC, Lin DT, Lin K-H. Epstein-Barr virus associated post-transplantation lymphoproliferative disorder with hemophagocytosis in a child with Wiskott-Aldrich syndrome. Pediatr Blood Cancer 2005;45:340–3. https://doi.org/10.1002/pbc.20191.

[6] Yoshida K, Minegishi Y, Okawa H, Yata J, Tokoi S, Kitagawa T, et al. Epstein-Barr Virus-associated malignant lymphoma with macroamylasemia and monoclonal gammopathy in a patient with Wiskott-Aldrich syndrome. Pediatric Hematology and Oncology 1997;14:85–9. https://doi.org/10.3109/08880019709030889.

[7] Avila-Smirnow D, Córdova-Aguilera M, Cantillano-Malone C, Arriaza-Ortiz M, Wegner-Araya A. Síndrome de Guillain Barré e hidrocefalia en un lactante con Síndrome de Wiskott Aldrich. Rev Chil Pediatr 2020;91:105. https://doi.org/10.32641/rchped.v91i1.1208.

[8] Olmez A, Turul T, Tezcan I, Turanli G. Guillain-Barré syndrome in an immunocompromised patient with Wiskott-Aldrich syndrome. Turk J Pediatr 2008;50:412–3.

[9] Xie V, Kornbluh A. Myelin oligodendrocyte glycoprotein antibody-associated disease (MOGAD) as a novel presentation of CNS autoimmunity in a pediatric patient with Wiskott-Aldrich syndrome (WAS). Neurology 2022;93:S18. https://doi.org/10.1212/01.wnl.0000903616.08384.e6.

[10] Model LM. Primary reticulum cell sarcoma of the brain in Wiskott-Aldrich syndrome. Report of a case. Arch Neurol 1977;34:633–5. https://doi.org/10.1001/archneur.1977.00500220067012.

[11] Yasuda Y, Yabe H, Inoue H, Shimizu T, Yabe M, Yogo Y, et al. Progressive multifocal leukoencephalopathy after allogeneic bone marrow transplantation for Wiskott–Aldrich syndrome. Pediatrics International 2008;50:238–40. https://doi.org/10.1111/j.1442-200X.2008.02549.x.

[12] Katz DA, Berger JR, Hamilton B, Major EO, Post MJ. Progressive multifocal leukoencephalopathy complicating Wiskott-Aldrich syndrome. Report of a case and review of the literature of progressive multifocal leukoencephalopathy with other inherited immunodeficiency states. Arch Neurol 1994;51:422–6. https://doi.org/10.1001/archneur.1994.00540160128016.

[13] Hutter JJ, Jones JF. Results of a thymic epithelial transplant in a child with Wiskott-Aldrich syndrome and central nervous system lymphoma. Clinical Immunology and Immunopathology 1981;18:121–5. https://doi.org/10.1016/0090-1229(81)90015-5.

[14] Vignesh P, Suri D, Rawat A, Lau YL, Bhatia A, Das A, et al. Sclerosing cholangitis and intracranial lymphoma in a child with classical Wiskott-Aldrich syndrome. Pediatr Blood Cancer 2017;64:106–9. https://doi.org/10.1002/pbc.26196.

[15] Sero L, Okur N, Yalcin AD, Unal A. Severe antenatal intraventricular hemorrhage in a newborn with WASP pathogenic variant. European Journal of Medical Genetics 2022;65:104553. https://doi.org/10.1016/j.ejmg.2022.104553.

[16] Gaspoz JM, Waldvogel F, Cornu P, Gugler E, Dayer JM. Significant and persistent improvement of thrombocytopenia after splenectomy in an adult with the Wiskott-Aldrich Syndrome and intra-cerebral bleeding. Am J Hematol 1995;48:182–5. https://doi.org/10.1002/ajh.2830480308.

[17] Kreetapirom P, Hongeng S, Manuyakorn W, Anurathapan U, Pakakasama S, Sirachainan N, et al. Successful HLA haploidentical HSCT with post-transplant cyclophosphamide in Wiskott-Aldrich syndrome. Bone Marrow Transplant 2017;52:913–4. https://doi.org/10.1038/bmt.2017.25.

[18] Conyers RK, Cole TS. Successful second bone marrow transplantation in a Wiskott-Aldrich syndrome patient with systemic vasculitis. J Allergy Clin Immunol 2016;137:1615–6. https://doi.org/10.1016/j.jaci.2015.11.008.

[19] Rai V, Sharma S, Singh A, Mehndiratta S, Chopra N. The misleading normal in an unusual case of Wiskott-Aldrich syndrome: a case report with review of literature. Indian J Med Paediatr Oncol 2025. https://doi.org/10.1055/s-0045-1805089.

[20] Ahuja A, Yanamandra U, Kapoor R, Chatterjee T. Unusual infection in a haploidentical transplant of Wiskott - Aldrich syndrome. Med J Armed Forces India 2023;79:729–31. https://doi.org/10.1016/j.mjafi.2023.08.006.

[21] Matsushima T, Nakamura K, Oka T, Tachikawa N, Sata T, Murayama S, et al. Unusual MRI and pathologic findings of progressive multifocal leukoencephalopathy complicating adult Wiskott-Aldrich syndrome. Neurology 1997;48:279–82. https://doi.org/10.1212/WNL.48.1.279.

[22] Downes SM. Visual loss due to progressive multifocal leukoencephalopathy in a congenital immunodeficiency disorder. Arch Ophthalmol 2001;119:1376. https://doi.org/10.1001/archopht.119.9.1376.

[23] Heidelberger KP, LeGolvan DP. Wiskott-Aldrich syndrome and cerebral neoplasia: report of a case with localized reticulum cell sarcoma. Cancer 1974;33:280–4. https://doi.org/10.1002/1097-0142(197401)33:1%3C280::AID-CNCR2820330141%3E3.0.CO;2-H.

[24] Sun Y, Song X, Pan H, Li X, Sun L, Song L, et al. Wiskott-Aldrich syndrome: a new synonym mutation in the WAS gene. Intractable Rare Dis Res 2024;13:69–72. https://doi.org/10.5582/irdr.2023.01102.

[25] Harfi HA, Al-Malik S, Tulba A. Wiskott-aldrich syndrome. ANN SAUDI MED 1992;12:355–61. https://doi.org/10.5144/0256-4947.1992.355.

[26] Cheminant M, Mahlaoui N, Canioni D, Desconclois C, Hermine O, Pasquet M, et al. Lymphoproliferative disease in Wiskott-Aldrich syndrome: analysis of the french national registry of primary immunodeficiencies. Blood 2014;124:4129. https://doi.org/10.1182/blood.V124.21.4129.4129.
